# Performance enhancement of perovskite solar cells through plasmonic titanium nitride nanoparticles

**DOI:** 10.1038/s41598-026-37468-0

**Published:** 2026-02-17

**Authors:** Mahmoud N. El-Mallah, Mostafa El-Aasser, Nasr Gad

**Affiliations:** Physics Department, Faculty of Science, Ain Shams University, Abbassia, Cairo, 11566 Egypt

**Keywords:** Perovskite solar cells (PSCs), Plasmonics, Absorption enhancement, CH_3_NH_3_PbI_3_, Refractory metals, TiN, Energy science and technology, Materials science, Nanoscience and technology, Optics and photonics, Physics

## Abstract

This work addresses enhancing the performance of Perovskite solar cells by incorporating ellipsoid plasmonic nanoparticles of Titanium Nitride (TiN) into the pure methylammonium lead iodide (CH_3_NH_3_PbI_3_) active layer. The device structure consists of ITO/TiO_2_/CH_3_NH_3_PbI_3_/PEDOT: PSS/Au. The study is conducted using finite difference time domain method for the optical studies and SCAPS-1D software for the electrical parameters, the proposed structure managed to boost the absorption of the perovskite solar cell to levels more than 90% in the visible and near infrared range (NIR), and a broad absorption band from 400 nm to 1200 nm is obtained, which is reflected as an electrical performance with short circuit current density of 46.84 mA/ cm^2^, and open circuit voltage of 0.8924 V, a fill factor of 76.22%, and an impressive power conversion efficiency of 31.8%. These results showcase the plasmonic potential of TiN refractory metal nanoparticles in enhancing the performance of perovskite solar cells.

## Introduction

With increasing concerns over climate change, photovoltaics (PV) play a crucial role in the global transition to cleaner and more sustainable energy sources. PV involves the conversion of light into usable electrical energy through PV cells, more often called solar cells (SCs). Considering the sun provides more than 10,000 times the current annual global energy consumption, researchers and engineers have focused on improving photovoltaic technology to make it more efficient, cost-effective, and widely applicable^1^.

Perovskite solar cells (PSCs) are a third-generation SC that have emerged rapidly as a leading technology in PV, due to performance improvements that have been observed in recent years. These cells have shown efficiency levels in converting light into electricity comparable to well-established photovoltaic technologies like silicon^2,3^. These massive advancements have gotten the deserved consideration of both academics and professionals alike, making PSCs a key stone in the renewable energy field. PSCs have numerous unique properties, from a broad absorption spectrum to high power conversion efficiencies, long diffusion lengths, relatively simple synthesis processes, and low manufacturing costs^4–7^, making PSCs an attractive candidate for future SC technologies.

Methylammonium lead iodide (CH_3_NH_3_PbI_3_) perovskite, in particular, has been extensively studied for SC applications because of its direct bandgap of ~ 1.55 eV, which is very close to the ideal for the Shockley-Queisser optimal bandgap (~ 1.34 eV) for single-junction SC^4,8–13^. CH_3_NH_3_PbI_3_ absorbs strongly in the visible spectrum; its absorption coefficient decreases for wavelengths longer than 750 nm, limiting harvesting of the near-infrared portion of the solar spectrum^14^. To meet the ever-increasing energy demand, further improvements in light harvesting and charge collection are crucial.

Plasmonics is the collective oscillations of free electrons localized at the surfaces of metallic nanostructures (usually Ag, Au, or Al)^15–21^. The induced electric charge fluctuations result in electromagnetic oscillations. At resonance, the electric field of the incident electromagnetic waves drives the collective oscillations of the electrons in the metal to create a strong charge displacement and field concentration. This coupling between the incident electric field and the electrons is called localized surface plasmons (LSPs)^22–25^. LSPs are often higher than the exciting fields and located at the plasmonic material surface. This amplified field enhances light absorption and the scattering cross section for the incident electromagnetic waves, effectively increasing the optical path of the light^26^. Furthermore, it creates a strong near field in the proximity of the metal particle surface, which in turn improves light absorption in the SC active absorbing layer, enhancing the performance of the device^27^. Refractory metals like Titanium (Ti) are known for their high melting points and their capability to work normally at higher temperatures than other metals, making them more suitable materials as solar absorbers. Studies have shown that metamaterials made of Ti and its composites have a wide absorption spectrum and strong plasmon behavior. Moreover, Ti is more abundant in Earth’s crust than traditional precious metals such as gold and silver, which can lower the cost^28–34^.

In this study, ellipsoid plasmonic nanoparticles based on titanium nitride as a refractory material are introduced with rectangular and hexagonal arrangements. The absorber layer based on CH_3_NH_3_PbI_3_ is studied with/without the plasmonic nanoparticles. The absorptance was calculated in the wavelength range from 300 to 1500 nm, in the case of PSC with plasmonic nanoparticles, a broadband from 400 to 1200 nm having an absorption ≥ 90% is obtained compared to the basic perovskite without the nanoparticles, which achieved a relatively narrow band from 400 to 750 nm. The electrical parameters obtained are as follows: the short-circuit current density (J_sc_) is 46.84 mA/cm², the open-circuit voltage (V_oc_) is 0.8924 V, the fill factor (FF) is 76.22%, and the power conversion efficiency (PCE) is 31.8%.

## Simulation and methodology

**Fig. 1:**
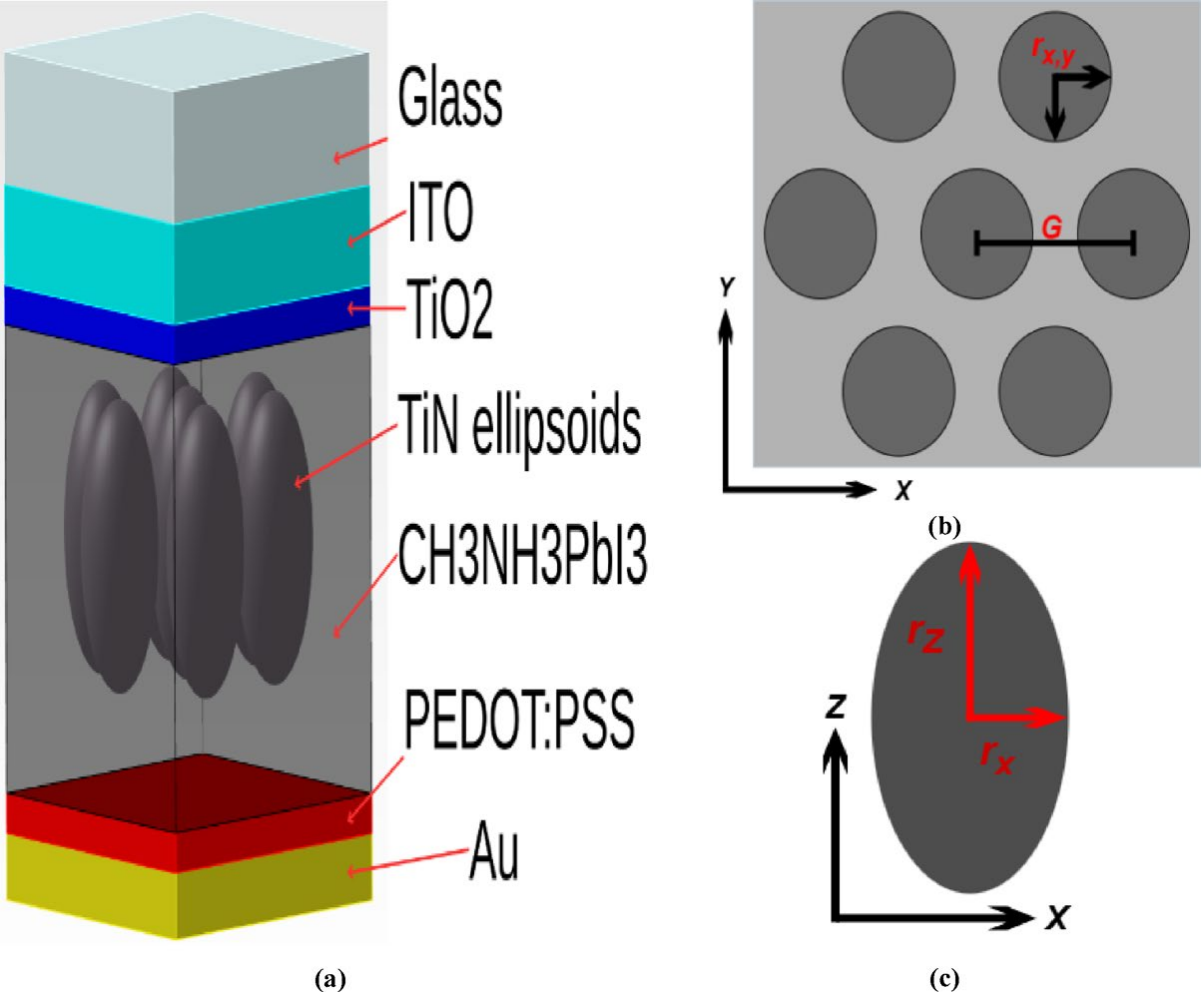
**(a)** 3 d view of the proposed structure, **(b)** top-view of the CH_3_NH_3_PbI_3_ absrober layer with the ellipsoid nanoparticles, **(c)** side-view of the TiN ellipsoid.

This research focuses on the study of optical behavior using a simulation based on FDTD and electrical outcomes by the SCAPS simulator. The main objective was to assess the impact of the integrated nanoparticles on the device’s performance to offer insights that enable the development of more efficient PSCs. The proposed device structure, as shown in Fig. 1 started as a 400 (x) nm * 400 (y) nm, normal (n–i–p) SC structure with the configuration of: indium tin oxide (ITO) layer as a transparent conducting front electrode (TCO), TiO_2_ as both an electron transport layer (ETL) as well as an anti-reflection coating^35^, CH_3_NH_3_PbI_3_ as the perovskite absorber layer, poly(3,4-ethylenedioxythiophene) polystyrene sulfonate (PEDOT: PSS) as a hole transport layer (HTL)^36,37^, and an Au layer that acts as a back reflector to limit transmission as well as a back contact. To strengthen the local electromagnetic field, a hexagonal lattice array of ellipsoid Titanium Nitride (TiN) nanoparticles is integrated into the perovskite layer.

All is enclosed with a glass layer to provide support and protect the PSC from degradation over time due to exposure to moisture, oxygen, and UV light. The dimensions of every layer and the radii of the ellipsoids are shown in Table 1.

**Table 1:** Dimensions of the design.

Layer	Thickness (z) (nm)
Glass	200
ITO	75
TiO_2_	30
CH_3_NH_3_PbI_3_	350
*PEDOT: PSS*	*30*
*Au*	*50*
*TiN ellipsoids (Optimal)*	*r*_*X, Y*_ *= 60*,* r*_*Z*_ *=110;* Fig. 1.*(c)*
*Distance from the center of the ellipsoids to the surface of the perovskite layer*	*155*
*Array constant G (Optimal)*	*140*
*The closest distance between any two neighboring ellipsoids (at their centers) = G − 2 r*_*X, Y*_	*20*

### Optical simulation

**Fig. 2:**
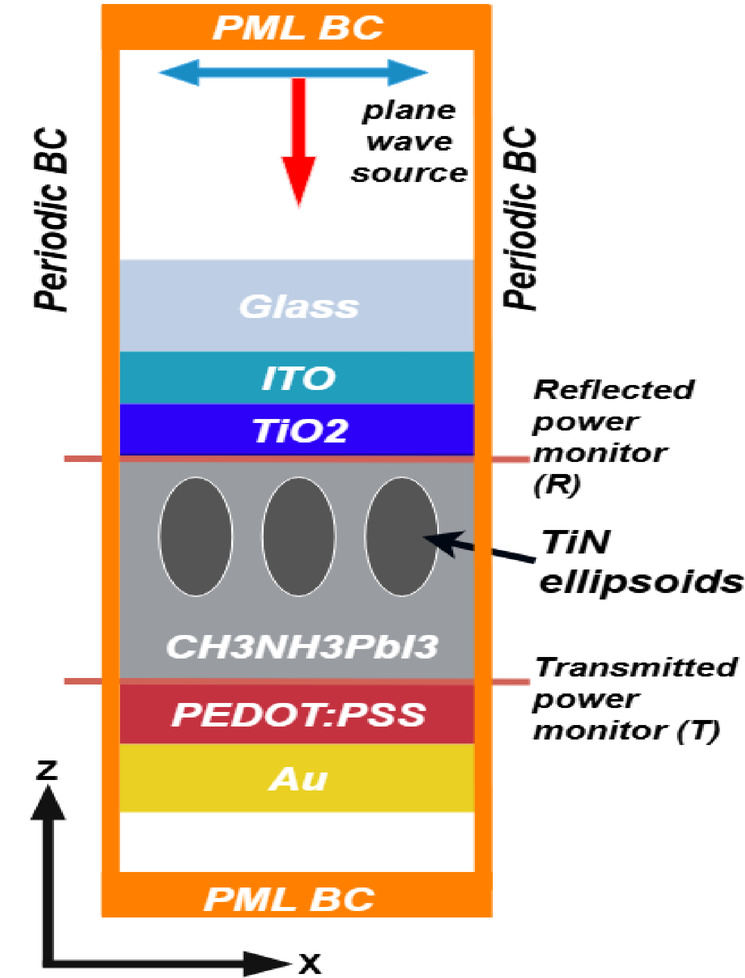
Optical simulation setup.

The optical simulation was carried out using the FDTD method, as shown in Fig. 2. A plane wave source is used with wavelength in the 300–1500 nm range, the structure variation is confined in the Z direction, with the X and Y directions being fully periodic, therefore periodic boundary conditions were applied to X and Y directions while perfectly matched layers boundary conditions (PML) were applied to the Z direction to prevent further reflections. Proper meshing was used to capture the fine changes of the electric field near the interfaces and to account for the continuous change of the ellipsoid surface, the meshing steps used were 3*3*2.5 nm and meshing convergence processes were executed to ensure that further meshing refinement is not necessary. The optical constants (n and k) of TiO_2_ were obtained from ref^38^., CH_3_NH_3_PbI_3_ from ref^39^., PEDOT: PSS data points extracted from supplementary materials (Figure S2) from ref^40^., TiN from ref^41^., ITO from ref^42^., Au from ref^43^. These wavelength-dependent values were directly imported into the FDTD simulation.

After performing the simulation, the used source was normalized to the standard AM1.5G (air mass solar spectrum), to ensure that the resulting optical generation profiles are accurately calculated across the relevant wavelength range of the source. Power monitors were used to calculate the reflectance (R) and transmittance (T), thus calculating the absorptance (A) using the relationship: **A(λ) = 1 - R - T.** The FDTD software calculates the optical generation as a 3-dimensional matrix using this expression^36,44^:

1 $$\:G\left(\overrightarrow{r}\right)=\frac{{P}_{\mathrm{abs}}\left(\overrightarrow{r},\omega\:\right)}{\hslash\:\hspace{0.17em}\omega\:}$$

where P_abs_ is the absorption spatial power density, and its expression is:

2 $$\:{P}_{\mathrm{abs}}\left(\overrightarrow{r},\omega\:\right)\mathrm{=}-0.5\hspace{0.17em}{\left|E\left(\overrightarrow{r},\omega\:\right)\right|}^{2}\hspace{0.17em}\mathrm{Im}\left[\epsilon\left(\overrightarrow{r},\omega\:\right)\right]$$

### Electrical simulation

Electrical simulations were conducted by SCAPS-1D, which as the name implies, is a one-dimensional simulation tool specifically designed for SCs, developed by the Department of Electronic and Information Systems (ELIS) at Ghent University^45–47^.

SCAPS-1D is based on three equations, Poisson’s (3), electrons (4), and holes (5) steady-state continuity equations:

3 $$\:\frac{d}{dx}\left(-\epsilon\:\left(x\right)\frac{d\psi\:}{dx}\right)=q\left[p\left(x\right)-n\left(x\right)+{N}_{d}^{+}\left(x\right)-{N}_{a}^{-}\left(x\right)+{p}_{t}\left(x\right)-{n}_{t}\left(x\right)\right]$$

4 $$\:\frac{d{p}_{n}}{dt}={G}_{p}-\frac{{(p}_{n}-{p}_{n0})}{{\tau\:}_{p}}+{p}_{n}{\mu\:}_{p}\frac{d\xi\:}{dx}+{\mu\:}_{p}\xi\:\frac{d{p}_{n}}{dx}+{D}_{p}\frac{{d}^{2}{p}_{n}}{d{x}^{2}}\:$$

5 $$\:\frac{d{n}_{p}}{dt}={G}_{n}-\frac{\left({n}_{p}-{n}_{{p}_{0}}\right)}{{\tau\:}_{n}}\:+{n}_{p}\:{\mu\:}_{n}\:\:\frac{d\xi\:}{dx}+{\mu\:}_{n}\:\xi\:\:\frac{d{p}_{n}}{dx}+{D}_{n}\:\frac{{d}^{2}{n}_{p}}{d{x}^{2}}$$

Where *G*_*p*_ and *G*_*n*_ are the hole and electron generation rates, respectively. $\:{\tau\:}_{n}$ and $\:{\tau\:}_{p}$ are the lifetimes of electrons and holes, respectively; *D*_*p*_ and *D*_*n*_ are the hole and electron diffusion coefficients, respectively; $\:q$ is the charge of the electron; $\:\psi\:$ is the electrostatic potential; $\:{\mu\:}_{n}$ and $\:{\mu\:}_{p}$ are the mobilities of electrons and holes, respectively; $\:n\left(x\right)$ and $\:p\left(x\right)$ are free electrons and holes concentrations; $\:{p}_{t}\left(x\right)$ and $\:{n}_{t}\left(x\right)$ are the concentrations of trapped holes and electrons, respectively. $\:{N}_{d}^{+}\left(x\right)$ and $\:{N}_{a}^{-}\left(x\right)$ are the donor and acceptor concentrations, respectively; $\:\xi\:$ is the electric field; $\:\mathrm{x:}$is the direction along the thickness of the SC^48^.

Since SCPAS-1D software is only a one dimensional solver and the optical generation profiles obtained from the FDTD are 3D matrices, the 3D generation matrix is processed averaging the data over the lateral dimensions (x and y) to produce a 1D profile in the Z direction, hence we imported the obtained 1D profile into SCAPS-1D to measure the effect of the ellipsoid TiN plasmonic nanoparticles on the performance of the device. The workflow was integrated using MATLAB for data processing and analysis. This approach is much faster and requires less computational power than the computationally intensive 3D electrical simulations. Although this method does not capture edge effects and possible carrier dynamics in the lateral dimensions, it has a second-order impact on the performance since the carrier transport behavior in thin-film SCs is predominantly in the vertical direction. The electrical properties of the materials and the recombination parameters, as shown in Table 2 were obtained from the literature^49–51^.

**Table 2:** Electrical parameters used for electrical simulation^49–51^.

Parameter	ITO	TiO_2_	CH_3_NH_3_PbI_3_	PEDOT: PSS
Thickness (nm)	75	30	350	30
Band gap Eg (eV)	3.5	3.2	1.5	1.6
Electron affinity, χ (eV)	4	3.9	3.9	3.4
Relative dielectric permittivity, ε_r_	9	9	30	3
CB-effective density of states, N_C_ (1/cm^3^)	2.2 × 10^18^	1 × 10^19^	2.5 × 10^20^	2.2 × 10^18^
VB-effective density of states, N_V_ (1/cm^3^)	1.8 × 10^19^	1 × 10^19^	2.5 × 10^20^	1.8 × 10^19^
Electron mobility, µ_n_ (cm^2^/Vs)	20	20	50	4.5 × 10^–2^
Hole mobility, µ_h_ (cm^2^/Vs)	10	10	50	4.5 × 10^–2^
Shallow-uniform acceptor density, N_A_ (1/cm^3^)	0	0	1 × 10^17^	1 × 10^18^
Shallow-uniform donor density, N_d_ (1/cm^3^)	1 × 10^21^	1 × 10^16^	0	0
Defect density, N_t_ (1/cm^3^)	1 × 10^15^	1 × 10^15^	1 × 10^13^	1 × 10^15^

## Results and discussion

**Fig. 3:**
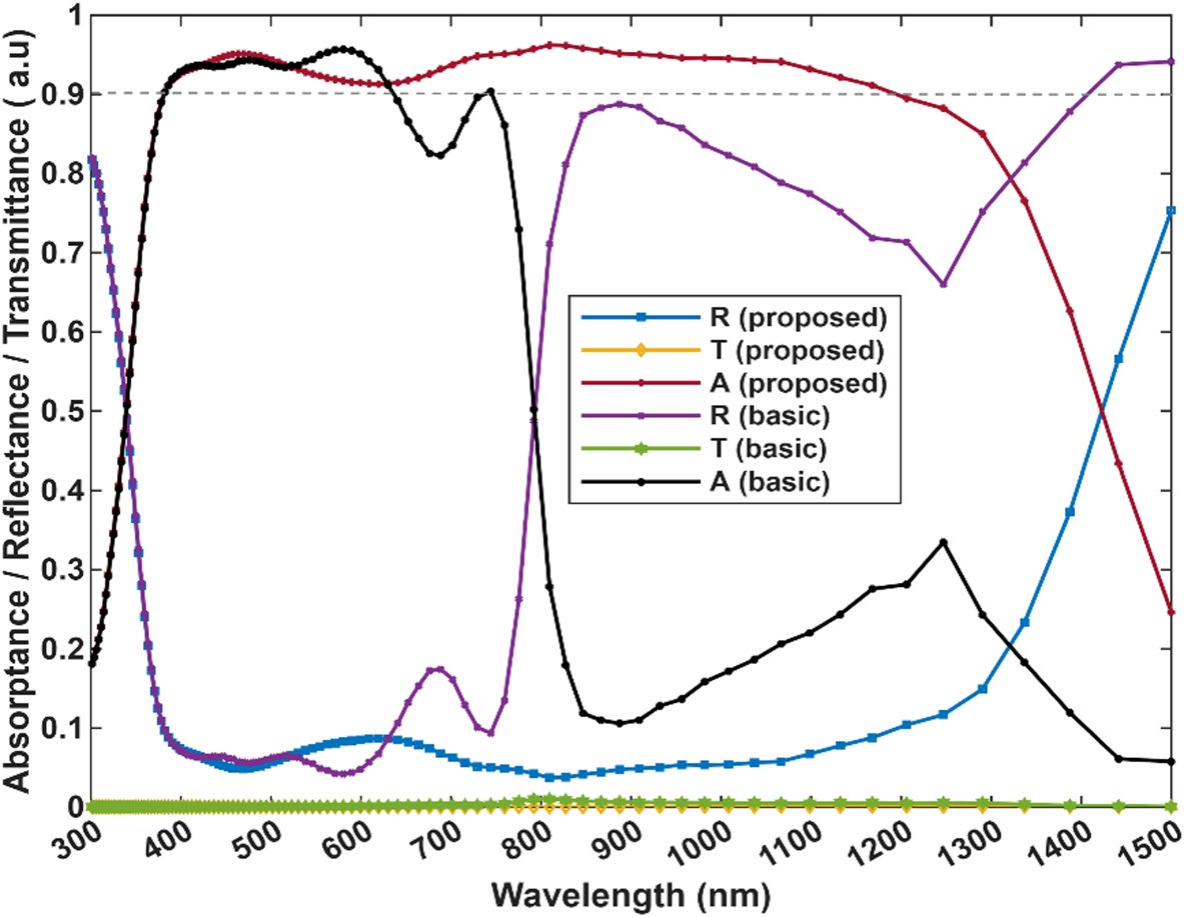
Spectral characteristics (A, R, T) curves of the proposed design vs. the basic perovskite cell without nanoparticles.

The proposed structure exhibits a broad absorption spectrum < 0.9 from 400 to 1200 nm (as shown in Fig. 3), with the remaining parts of the spectrum having solid performance as well, indicating that almost all of the solar spectrum is being utilized in the optical generation, Titanium nitride (TiN) nanoparticles are strong broadband absorbers with absorption from the visible range into the near-infrared (NIR) region, even though they typically have their primary plasmonic resonance in the visible range around 450 nm. Broadband absorption in TiN is due to the combination of several factors; TiN has unique dielectric properties that enable an extended and red-shifted localized surface plasmon resonance (LSPR), the absorption tails can extend well past 800 nm^52^. Modifying the shape of TiN nanostructures like dimers, nanocones, or disordered metasurfaces and in our case nano-ellipsoids can also widen and enhance the absorption spectrum, improving light trapping and resonance coupling over a large range of wavelengths^53–55^. The effect of the plasmonic TiN ellipsoids is clearly evident in maintaining high absorption in wavelengths longer than 750 nm meanwhile in the control structure, without the TiN ellipsoids, the absorption drops to an average of 0.25 beyond the 750 nm mark. The role of the gold back contact is shown in maintaining zero transmission across the entire spectrum and therefore A = 1-R. In the following analysis, the absorptance (A) will only be considered for convenience.

.

**Fig. 4:**
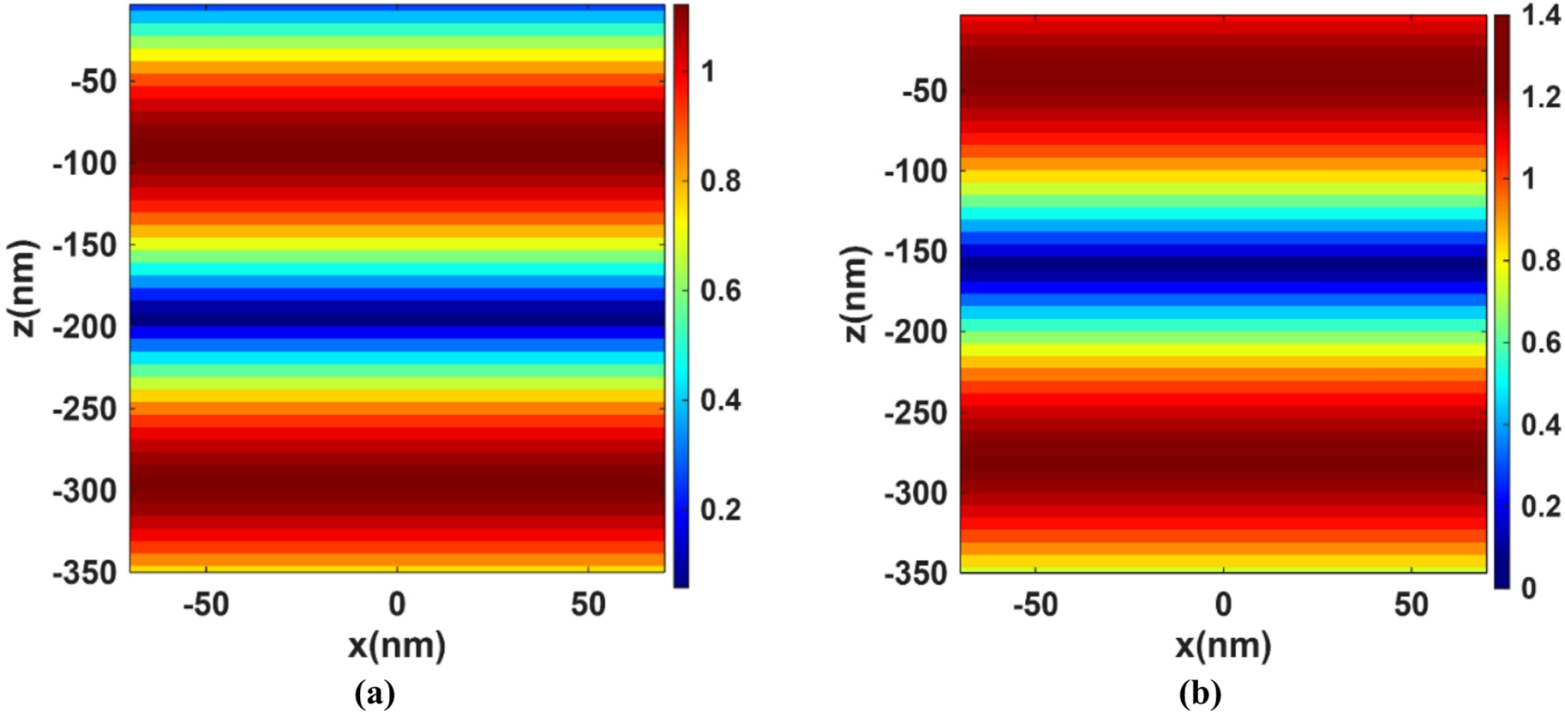
The solar radiation normalized electric field intensity |E|² (a.u.) for the basic perovskite at **(a)** 800 nm and **(b)** 1100 nm.

**Fig. 5:**
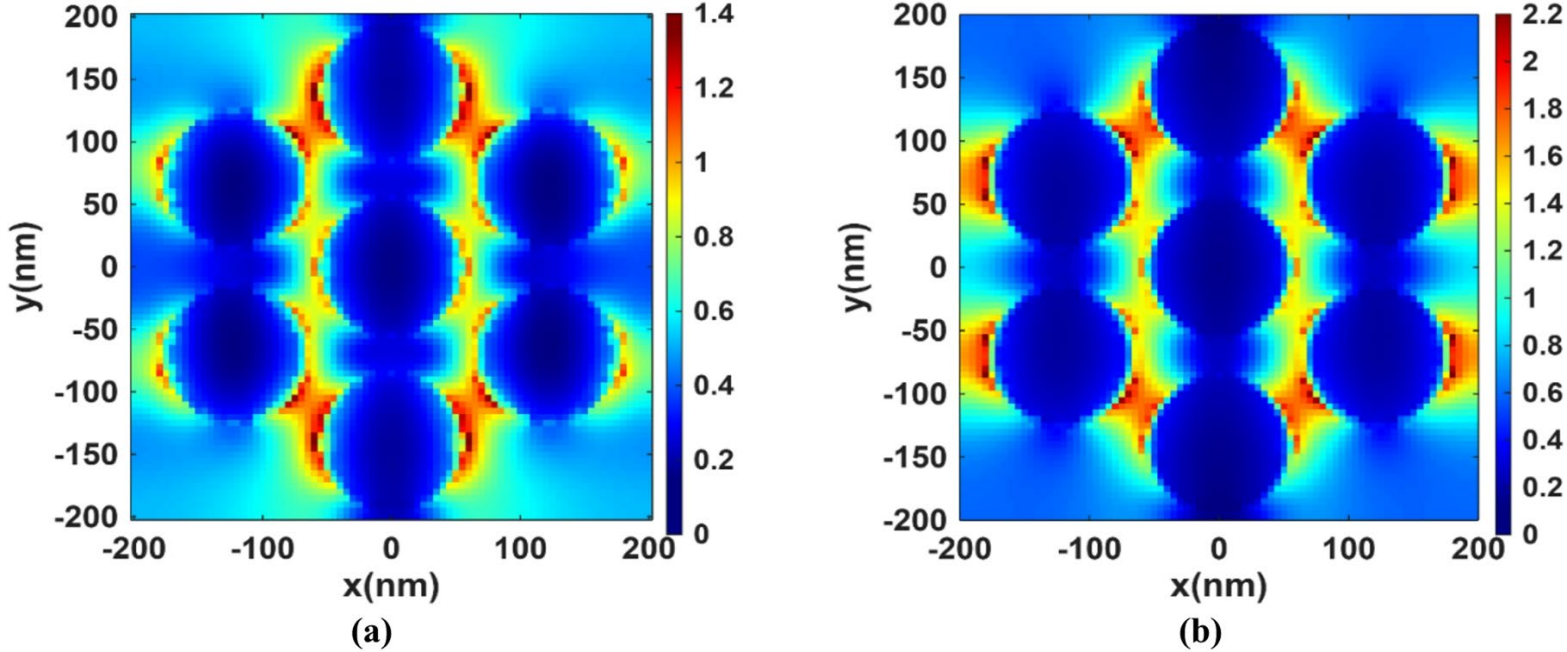
The XY-profiles of the normalized electric field intensity |E|² (a.u.) at **(a)** 800 nm and **(b)** 1100 nm.

**Fig. 6:**
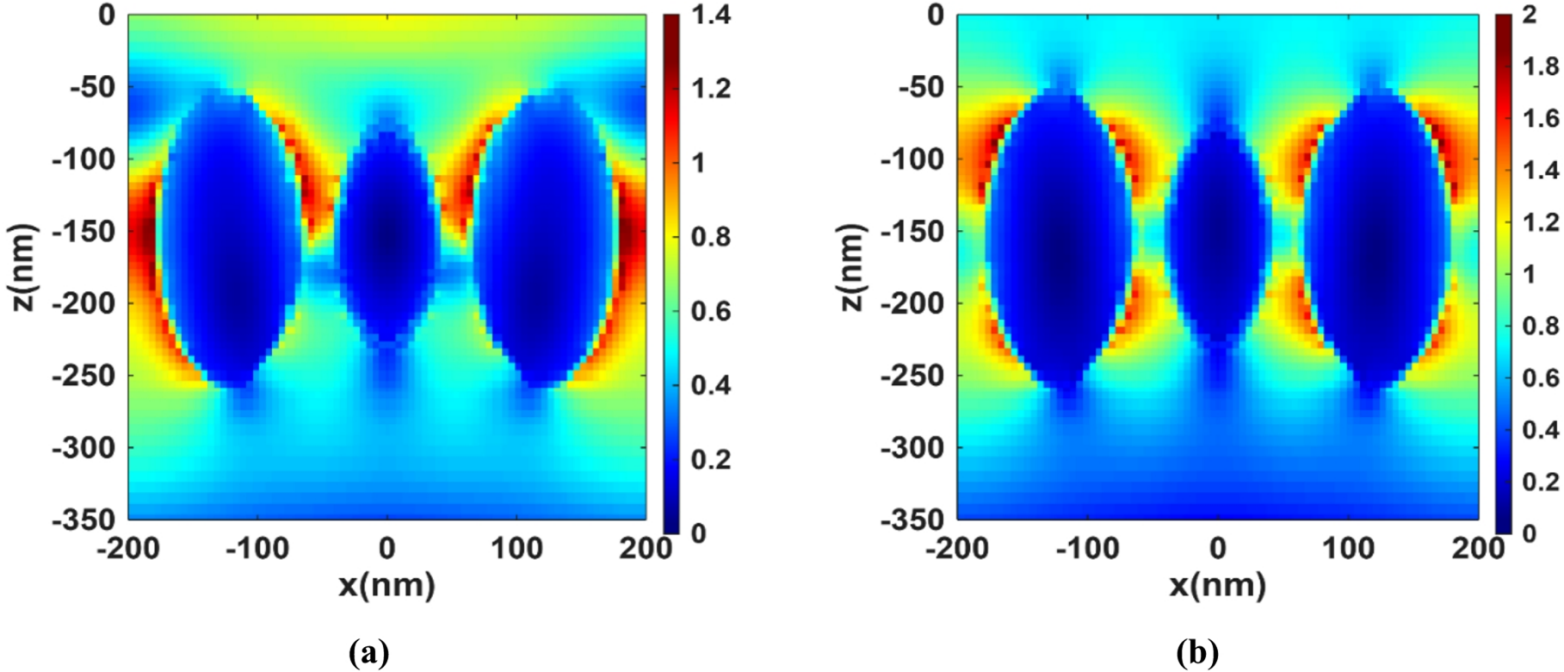
XZ-profile of the normalized electric field intensity |E|² (a.u.) for the proposed structure at **(a)** 800 nm and **(b)** 1100 nm.

Figure 4 shows the solar radiation going through the basic perovskite layer at wavelengths of (a) 800 nm and (b) 1100 nm. The color maps in Fig. 4 represent the normalized electric field intensity |E|² (a.u.) inside the perovskite absorber layer, obtained from the field profile in the FDTD simulation. Dark blue corresponds to regions of negligible field intensity, i.e., minimal absorption while value > 1 indicates field enhancement. In the absence of plasmonic nanoparticles, the radiation remains largely unchanged with minimal absorption. This behavior can be attributed to the reduction in the material’s absorption coefficient at longer wavelengths within the near-infrared (NIR) region, which accounts for the observed decline in absorption beyond 750 nm. At longer wavelengths, the perovskite’s absorption is weak, so the light penetrates the active layer and reflects from the Au back contact. These reflections create standing waves. At an antinode of a standing wave, the electric field amplitude can exceed the incident amplitude due to interference which explains observing values of 1.2 or more in the basic perovskite. Figure 5 presents the XY-profile at two wavelengths (a) 800 nm and (b) 1100 nm for the structure with integrated TiN nanoparticles, whose plasmonic effect leads to the excitation of LSPs, which enhances the electric field near the nanoparticles’ surfaces. This results in a stronger interaction between the incident light and the perovskite active layer and hence improved light absorption, particularly in the NIR range, where the basic perovskite lacks. Figure 6 extends the analysis to the XZ-plane at the same two wavelengths, this cross-sectional view further confirms the effect of the TiN nanoparticles on enhancing the local electromagnetic fields in the NIR region.

To further investigate our design, a parameter sweep analysis is applied to all key parameters like shape, size, and material of the nanoparticles.

**Fig. 7:**
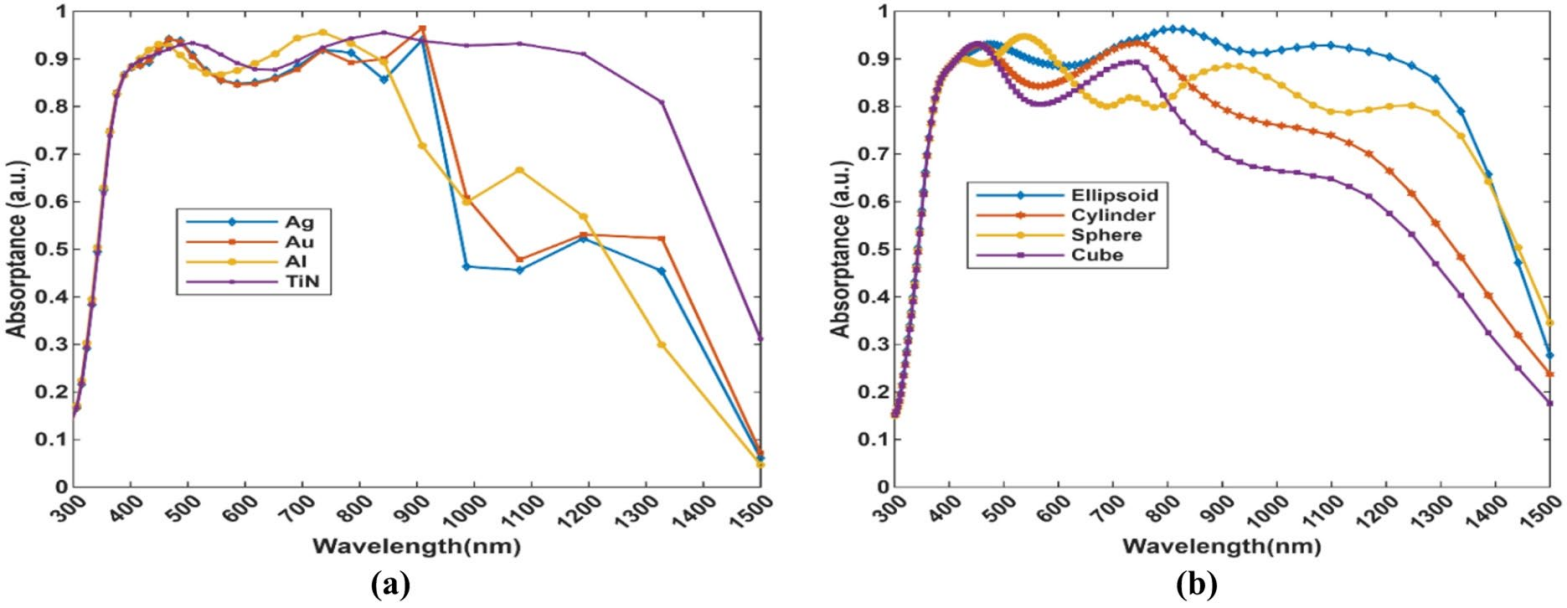
Absorptance spectra of different **(a)** materials for the ellipsoid nanoparticles and **(b)** shapes of the plasmonic TiN nanoparticles.

Figure 7.(a) illustrates the effect of the various common plasmonic materials such as gold (Au), silver (Ag), and aluminum (Al) against TiN. With its high melting point and strong plasmonic behavior as a refractory metal, TiN demonstrates superior absorption, especially in the NIR region, in comparison to the other common plasmonic materials, while being more cost-effective. In Fig. 7.(b), the effect of the nanoparticle shape on absorption is observed. The absorption by ellipsoids is higher across visible and NIR wavelengths than for both the spheres and cylinders. The difference can be explained by the excitation of both transverse and longitudinal modes in the ellipsoids due to its geometric properties that combine the advantages of both the curved surface of spheres and longitudinal length of cylinders in one shape, which in turn enhances light trapping and plasmonic coupling in the NIR region.

**Fig. 8:**
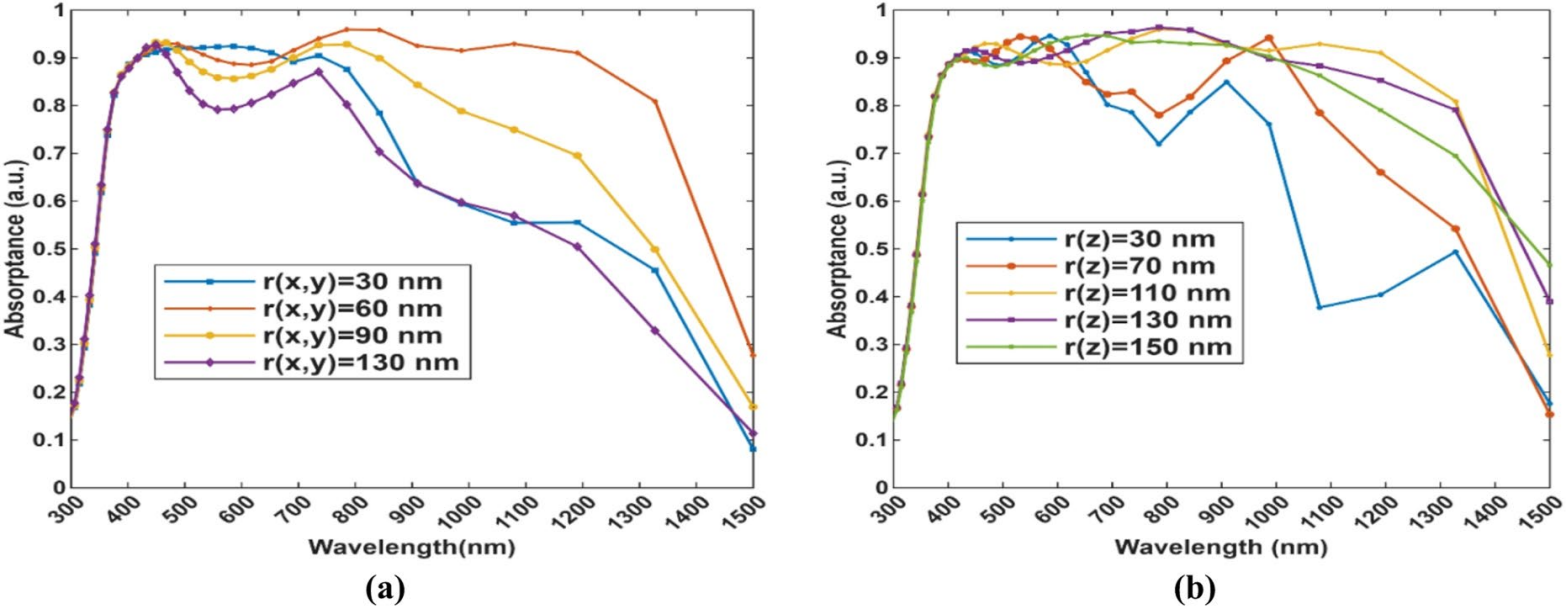
Absorptance spectra of **(a)** (x, y) radius of the TiN ellipsoids and **(b)** r(z) of the ellipsoids (radius in the direction of the thickness of the cell).

The effect of various radius values in the x, y, and z directions can be noticed, from Fig. 8.(a). r(x, y) = 60 nm is found to be the optimal value for maximizing the absorptance, while for r(z) as shown in Fig. 8.(b), the values of 110 nm and 130 nm are quite close, but 110 nm has a slightly higher average absorptance across the whole spectrum. Changes in the ellipsoid dimensions can affect the local field enhancement and the overall absorptance, emphasizing that precision in choosing the nanoparticle dimensions is necessary for optimizing the device performance.

**Fig. 9:**
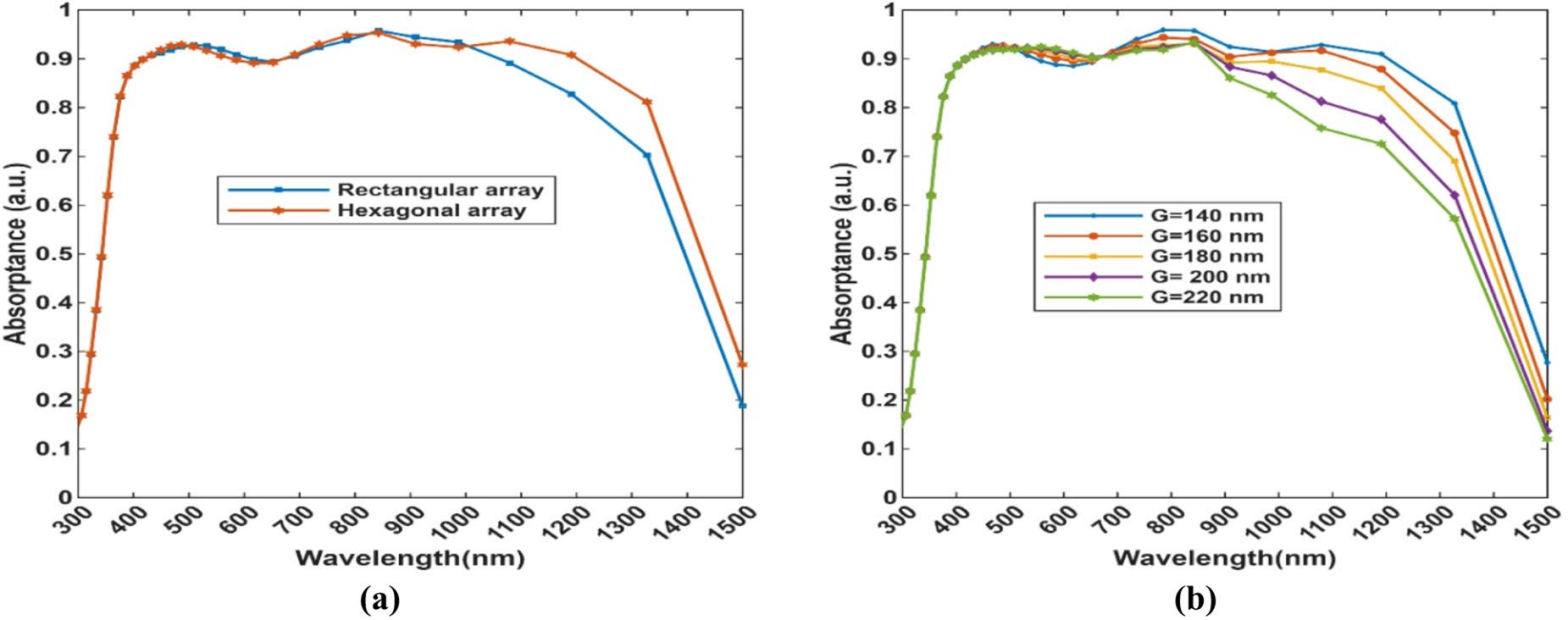
**(a)** Absorptance spectra of rectangular and hexagonal arrays and **(b)** absorption spectra with different values of hexagonal array constant (G) with 140 nm as the optimal value.

Figure 9.(a) compares hexagonal and rectangular arrays, such that between 400 and 1000 nm, absorptance is nearly saturated. However, the hexagonal array has higher absorption at wavelengths < 1000 nm. This is due to higher packing density and the 6-fold symmetry of the hexagonal lattice, which enables this type of arrangement to support stronger plasmonic resonances and field localization. Figure 9.(b) shows the effect of different values of lattice constant (G). Array constant (G) affects the periodicity of the nanostructure, which changes the surface plasmon polariton (SPP) coupling efficiency and therefore the photonic band gap position, leading to gradual degradation of coupling efficiency and field enhancement as G increases, particularly at wavelengths greater than 800 nm. The absorption is maximum at G = 140 nm, which in turn increases the pitch of the structure from 400 (x) nm* 400 (y) nm to 420 nm *420 nm to maintain the periodicity of the structure.

**Fig. 10:**
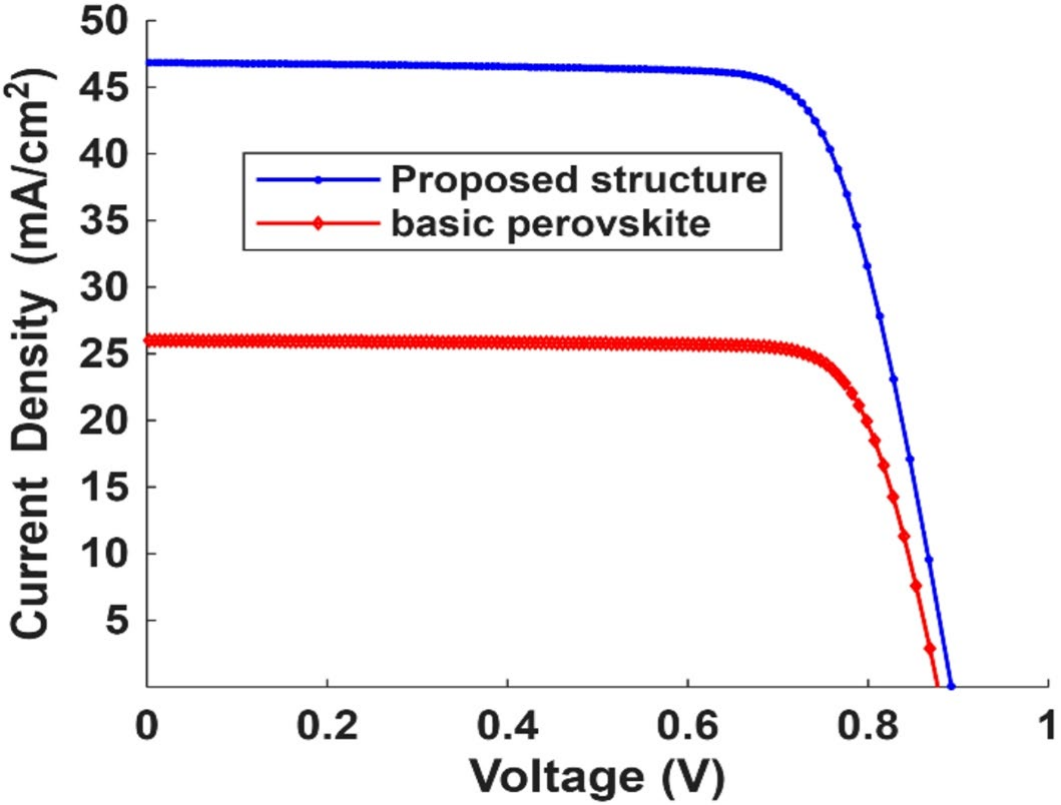
J-V curves for the proposed structure and the basic structures.

The obtained optical generation profile was then averaged over the lateral dimensions (x and y), then imported to SCAPS-1D, and after considering the defects of every layer and the interfaces between the different layers and recombination losses, we obtained impressive results of:

V_oc_ = 0.8924 V, J_sc_= 46.84 mA/cm^2^, Fill factor (FF) = 76.02% and power conversion efficiency (PCE) = 31.8%, very close to the Shockley–Queisser theoretical limit. Figure 10 shows the J-V curves obtained from SCAPS-1D for the basic perovskite material with and without the TiN refractory metals, which introduced a significant enhancement for PSC performance.

A comparison with the basic perovskite cell using the same methodology shows 80% enhancement in J_sc_ and 74.7% enhancement in PCE shown in Table 3 which indicates that the plasmonic enhancement of the absorption leads to a significant increase in optical generation and therefore the conversion efficiency.

**Table 3:** Electrical parameters simulation results.

Parameter	Basic perovskite	Proposed design	Δ%
V_oc_ (V)	0.877	0.8924	+ 1.75
J_sc_ (mA/cm^2^)	25.99	46.84	+ 80.02
V_mp_ (V)	0.74	0.7194	− 2.78
J_mp_ (mA/cm^2^)	24.72	44.293	+ 79.17
FF (%)	80.267	76.22	− 5
PCE (%)	18.2	31.8	+ 74.7

**Table 4:** Comparison of reported nanoparticle-enhanced PSCs with this work.

Reference	Nanoparticle Type & Structure	Integration/Location	PCE Improvement (Absolute/Relative)	Key Mechanism/Notes
^56^	Au@TiO_2_ core-shell nanospheres	TiO_2_ & perovskite layers	12.59% → 18.24% (44% ↑)	Enhanced exciton generation, charge separation
^57^	Triple core-shell (TiO_2_@Ag@TiO_2_, etc.)	MASnI_3_ absorber (simulated)	Up to 30.18% (64% ↑ vs. planar)	Light trapping, stability, optimal at 60 nm radius
^58^	Ag@SiO_2_, SiO2@Ag@SiO_2_ core-shell	Perovskite-compact TiO_2_ interface	14.83% → 19.72% (Ag@SiO_2_, 33% ↑)	Light trapping, reduced recombination
^59^	Au nanoparticles	SnO_2_ electron transport layer	10.96% → 13.84% (26% ↑)	Improved ETL conductivity, reduced recombination
^60^	Au nanoparticles (inkjet-printed)	TiO_2_ microdot array (ETL interface)	> 47% ↑ in PCE (relative)	Localized plasmon, improved crystallite size
This work	TiN ellipsoid nanoparticles	CH_3_NH_3_PbI_3_ absorber layer	18.2% → 31.8% (74.7%↑)	excitation of multiple plasmonic modes near-field scattering

To place our results in context, Table 4 represents this work compared to reported benchmark representative studies that embedded plasmonic nanoparticles into PSCs. The incorporation of plasmonic nanoparticles to date in PSCs has increased the performance but only reported moderate improvements in PCE (between 25 and 47%). For example, Au@TiO₂ core-shell nanospheres improved the PCE from 12.59% to 18.2% (≈ 44% increase) through additional exciton generation and charge separation^56^. The multilayer triple core-shell designs added to MASnI₃ absorbers achieved simulated efficiencies of 30.18% (≈ 64% increase) by absorbing more light and providing stability^57^. Similar to the previous 2 examples, Ag@SiO₂ and SiO₂@Ag@SiO₂ structures improved PCE from 14.8% to 19.7% (≈ 33% increase) through UV laser pumping through reduction of recombination^58^. The Au nanoparticles deposited at the ETL interfaces improved the subsequent performance of the device by improving conductance and crystallinity^59^. Compared to the previously mentioned benchmarks, the improvement delivered in our TiN ellipsoidal nanoparticle design has significantly larger relative increase in efficiency from 18.2% to 31.8% (≈ 74.7%). This is due to the combined contributions made in maximum carrier generation from working with anisotropic ellipsoids, which allows for multiple plasmonic modes to be excited, along with strong near-field scattering, giving rise to broadband absorption extending well into the NIR spectral region.

The results (V_oc_ = 0.892 V, J_sc_ = 46.84 mA/cm², FF = 76.0% and PCE = 31.8%) are very close to the Shockley–Queisser limit. While these results are more optimistic than most experimental studies described in the literature, it is important to remember that these results are based on idealized optical and electrical simulations. We did consider plasmonic enhancement and any shading due to the TiN nanoparticles. However, the net balance was clearly weighted towards light-harvesting improvement. In practical use, experimental results can vary and normally will be lower because of material quality and defects in fabrication, but our results demonstrate the theoretical capabilities of TiN ellipsoids for near-limit perovskite SCs efficiencies. These findings provide the researchers with a clear benchmark to build on and illustrate the strong potential of TiN ellipsoids for advanced PSCs, pushing the walls toward their theoretical limits.

## Conclusion

Throughout this work, we have shown using optical (FDTD) and electrical (SCAPS) simulations that embedding ellipsoid titanium nitride (TiN) plasmonic nanoparticles into perovskite solar cells can greatly improve the cell performance. Our key findings are:

- **Broadband Optical Enhancement**: The ellipsoid TiN nanoparticles excite multiple plasmonic resonances that enhance light absorption across a wide spectral range (400–1200 nm), maintaining the average absorptance above 0.9.
- **Improved Carrier Generation and Collection**: The optical enhancement results in a higher optical generation profile, when imported into SCAPS, yields a great increase in short circuit current density (J_sc_) from 25.99 mA/cm² to 46.84 mA/cm².
- **Improved Power Conversion Efficiency: **The power conversion efficiency improves from 18.2 % in the vanilla cell to 31.8 % in the plasmonic device, primarily because of the increase in J_sc_.
- **Parametric analysis: **A parameter sweep analysis indicated that the nanoparticle geometrical shape, dimensions, and lattice array can affect the optical and hence the electrical performance, with an optimal configuration that maximizes the absorption across the solar spectrum and hence higher generation rates and improved device conversion efficiency.

## Data Availability

The datasets generated and/or analyzed during the current study are available from the corresponding author upon reasonable request.
